# A Rare Case of Ramsey Hunt Syndrome With Cranial Polyneuropathy: Findings of the Brain MRI

**DOI:** 10.7759/cureus.75817

**Published:** 2024-12-16

**Authors:** Vasileios Papanikos, Nefeli Chaniotaki, Georgios Batsaouras, Iliana Chara Papanikou, Vasileios Kakouris, George D Oikonomou, Petros Zampakis

**Affiliations:** 1 Department of Otorhinolaryngology, Head and Neck Surgery, University Hospital of Patras, Patras, GRC; 2 Department of Radiology, University Hospital of Patras, Patras, GRC; 3 Department of Ear, Nose and Throat (ENT) and Audiology, St George's University Hospitals NHS Foundation Trust, London, GBR; 4 Department of Microbiology, University Hospital of Patras, Patras, GRC; 5 Department of Neurology, School of Medicine, University Hospital of Patras Greece, Patras, GRC; 6 Department of Interventional Neuroradiology, University Hospital of Patras, Patras, GRC

**Keywords:** cranial polyneuropathy, facial nerve palsy, meningoencephalitis, radiological diagnosis, ramsey hunt syndrome, vzv mri findings

## Abstract

In this case, we present the case of a 74-year-old female patient who visited the University Hospital of Patras, Greece, because of a 10-day history of earache and discharge in the left ear. Concurrently, the patient exhibited ipsilateral peripheral facial nerve palsy. We also observed vesicular eruption at the auricle and the external auditory canal (EAC) of the left ear. Following a thorough examination, the diagnosis of Ramsey Hunt syndrome (RHS) was established. Initial MRI scan of the head and neck indicated enhancement of both the left facial nerve (VII) and the vestibulocochlear (VIII) nerve, raising suspicion of RHS, which was subsequently confirmed through a positive polymerase chain reaction (PCR) test for varicella-zoster virus (VZV) DNA. The patient experienced a gradual clinical decline, ultimately affecting seven cranial nerves (CNs), with the emergence of meningoencephalitis. A follow-up brain MRI unveiled additional enhancement in CNs (VII, VIII, IX, XII) alongside signs of meningitis and encephalitis. Treatment included acyclovir administration (500 mg three times daily for one month), concurrent administration of prednisolone (125 mg per day for 20 days), permanent tracheostomy, and dedicated six-month physical rehabilitation. Over the course of a six-month follow-up, the patient exhibited significant clinical improvement and regained her ability to ambulate independently.

## Introduction

Varicella-zoster virus (VZV), a double-stranded DNA virus within the herpesvirus family, underlies two distinct clinical entities: chickenpox, typically presenting as the initial infection, and shingles, which emerges following viral reactivation at a later stage [1]. Immunodeficiency stands out as the principal predisposing factor for the reactivation of this virus [2]. The hallmark symptom of shingles is the development of a painful rash accompanied by blisters, and whenever it affects the auricle or/and the external auditory canal, it is called herpes zoster oticus (HZO) [3]. Moreover, VZV can lead to neurological complications by affecting the meninges, brain, and/or cranial nerves (CNs) [4,5]. Among these, facial nerve involvement is the most frequently observed, giving rise to a clinical entity known as Ramsay Hunt syndrome (RHS), which is mainly characterized by facial palsy, ear pain, and facial weakness [3]. Moreover, the vestibulocochlear nerve represents the second most involved cranial nerve in RHS, mainly causing hearing loss, while VZV infection can also lead to the onset of meningitis and encephalitis, which may have severe repercussions for the patient's health [4].

## Case presentation

A 74-year-old female patient attended the emergency department of our hospital for evaluation of a 10-day ear discharge from the left ear and severe ipsilateral otalgia. The patient also reported weakness and numbness on the same side of her face, which began several hours before she was admitted to the hospital. At the emergency department, she was afebrile and normotensive at 83 bpm/min. Her medical history revealed systematic lupus erythematosus (SLE) well controlled with hydroxychloroquine sulfate (200 mg per day) and methylprednisolone (10 mg daily), chronic arthritis, elevated blood pressure, glaucoma, and bilateral cataracts. Detailed history also revealed a tooth extraction within the last two weeks. The primary presenting symptom was left facial paralysis, which was classified as grade IV according to the House-Brackmann scale. Dropping of the left lower lip angle and concomitant inability to close the corresponding eye, eyelid ptosis, loss of forehead wrinkles, and left nasolabial fold confirmed the peripheral lesion of the CN (VII). Moreover, we observed a vesicular eruption on both the auricle and external auditory canal (EAC) of the left ear. The patient also exhibited balance disturbances and evoked nystagmus with a rapid beating phase directed towards the right side, without symptoms of tinnitus, nausea, or vomiting.

Diagnostic assessment

Initial laboratory examination demonstrated normal white blood cells at 10.94 cells/ml (4-11 cells/ml), lymphocytes at 8.4% (normal range: 20-40%), polymorphonuclear leukocytes (PMNs) at 82.3 % (normal range: 50-70%), and C-reactive protein (CRP) at 0.58 mg/dl (normal: <0.8). On the second day of hospitalization, vestibular disorders, hearing loss, and tinnitus also appeared (vestibulocochlear nerve - VIII). Although the tympanogram showed normal middle ear function, the audiogram indicated a unilateral sensorineural hearing loss of 30 dB at the higher frequencies of the left ear (4000 Hz and 8000 Hz). Weber and Rinne tests confirmed the sensorineural type of hearing loss, with sound lateralized to the unaffected right ear in the Weber test and positive Rinne findings on both sides (air conduction better than bone conduction). A subsequent brain MRI scan, performed on the fifth day, revealed enhancement of the cranial nerves VII and VIII, raising suspicion of VZV infection. Therefore, intravenous acyclovir (3 g, three times daily) was immediately administered to protect against shingles. In addition, serologic testing indicated anti-hepatitis B surface antibodies (anti-HBS-Abs) (+), anti-core immunoglobulin G antibodies​​​​​ (G-Abs) (+), hepatitis A virus antibody, immunoglobulin G (HAVAb IgG-Abs) (+), HIV antigen/antibody (Ag/Ab) (-), Epstein-Barr virus (EBV) (-), and cytomegalovirus (CMV) (-); a subsequent cerebrospinal fluid (CSF) culture was negative for VZV. A lumbar puncture was performed, and the results showed 65 cells per mm^3^, lymphocytosis with 53 lymphocytes, 6 PMNs, six monocytes, and both elevated glucose of 53 mg/dl and protein at 166,7 mg/dl. The findings suggested viral infection of the CNS. Cytology demonstrated lymphoid infiltrates, with positive immunocytochemical staining for CD3 T-lymphocytes and negative staining for L26 P-lymphocytes. Polymerase chain reaction (PCR) testing of the CSF was positive for VZV DNA and confirmed the diagnosis of herpes zoster virus and RHS.

The patient's condition deteriorated a week later with pharyngeal symptoms: absent gag reflex and pharyngeal paralysis with dysphagia (glossopharyngeal nerve - IX). Hence, a nasogastric tube was placed. Flexible nasal endoscopy (FNE) and oral examination revealed extensive aphthous ulcers in the oropharynx and larynx, involving the right faucial pillar, epiglottis, false vocal cords, and aryepiglottic fold on the right side, with salivary collection in the piriform sinuses. Increased respiratory secretions and fever occurred the next day. Further clinical examination revealed the weakness of the left sternocleidomastoid and ipsilateral trapezium muscle (accessory nerve - XI) impairment. Tongue deviation to the right side was observed when the tongue was extended, signifying hypoglossal nerve (XII) involvement (hypoglossal nerve - XII). Two days later, the patient complained of toothache with reflexes along the course of the trigeminal nerve and pain at the mandible (trigeminal nerve - V). An additional left hemi-laryngeal palsy was accompanied by hoarseness and cessation of the vomiting reflex (vagus nerve - X). FNE confirmed immobility of the left true vocal cord in both adduction and abduction positions, along with swollen aryepiglottic folds. Gradual but rapid upper airway obstruction led to tracheostomy after two days. Eventually, the patient developed meningoencephalitis, which was confirmed by a new brain MRI showing enhancement in CN (VII, VIII, IX, XII) along with signs indicative of meningitis and encephalitis.

MRI findings

**Figure 1:**
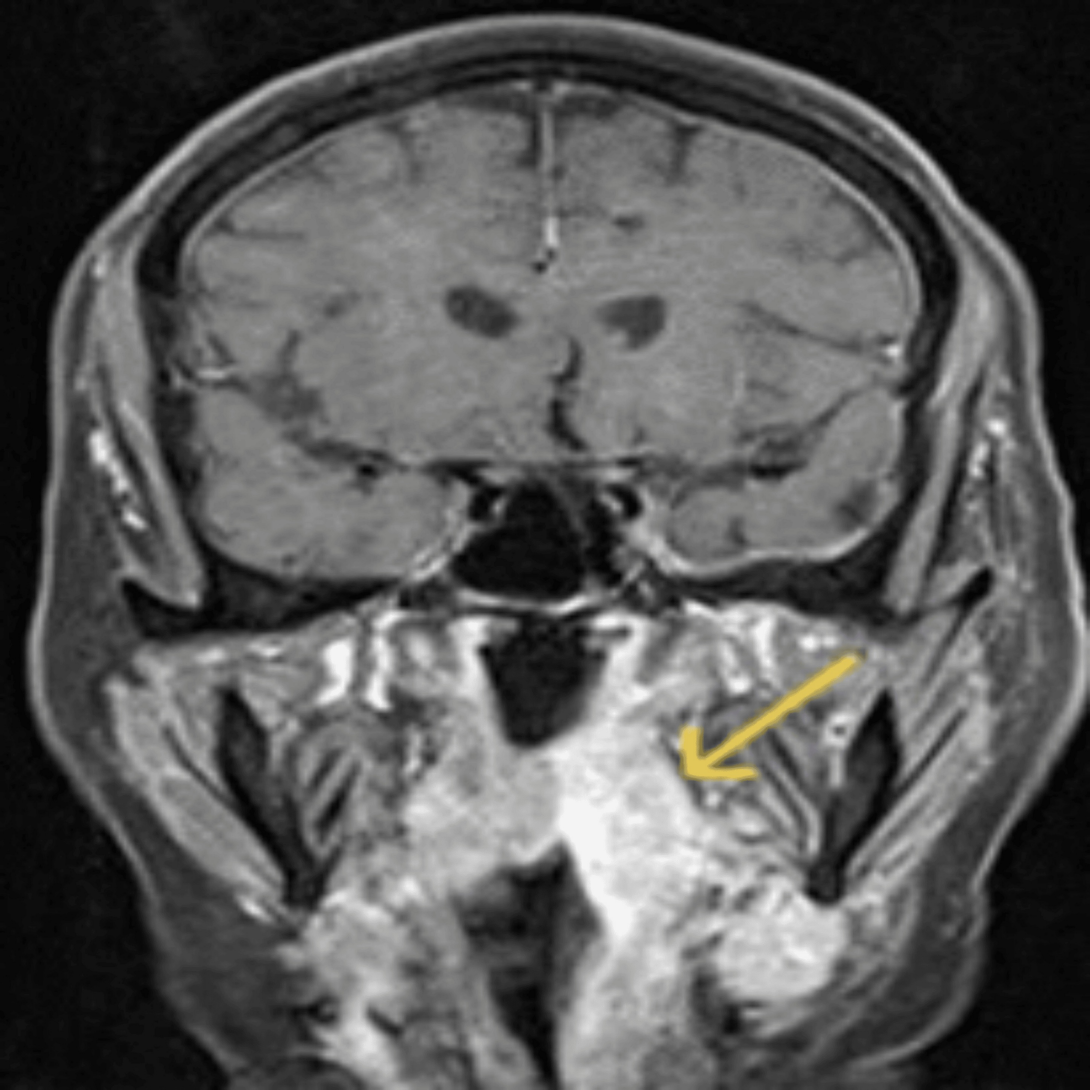
Coronal gadolinium-enhanced T1-weighted MRI The arrow indicates enhancement and thickening of the left-sided pharyngeal mucosal space.

**Figure 2:**
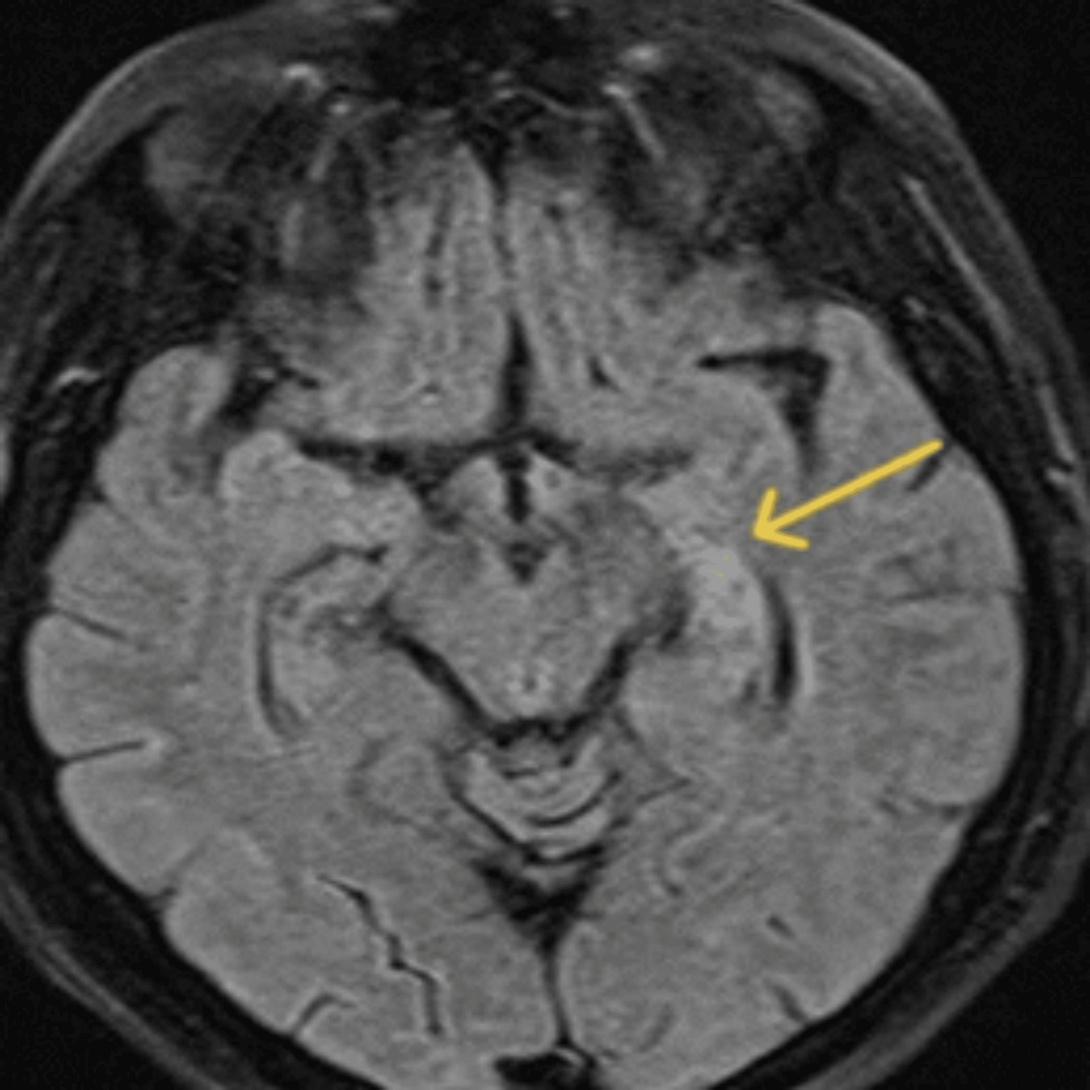
Axial T2-weighted FLAIR brain MRI The arrow shows a high signal area at the body of the left hippocampus. FLAIR - fluid-attenuated inversion recovery

**Figure 3:**
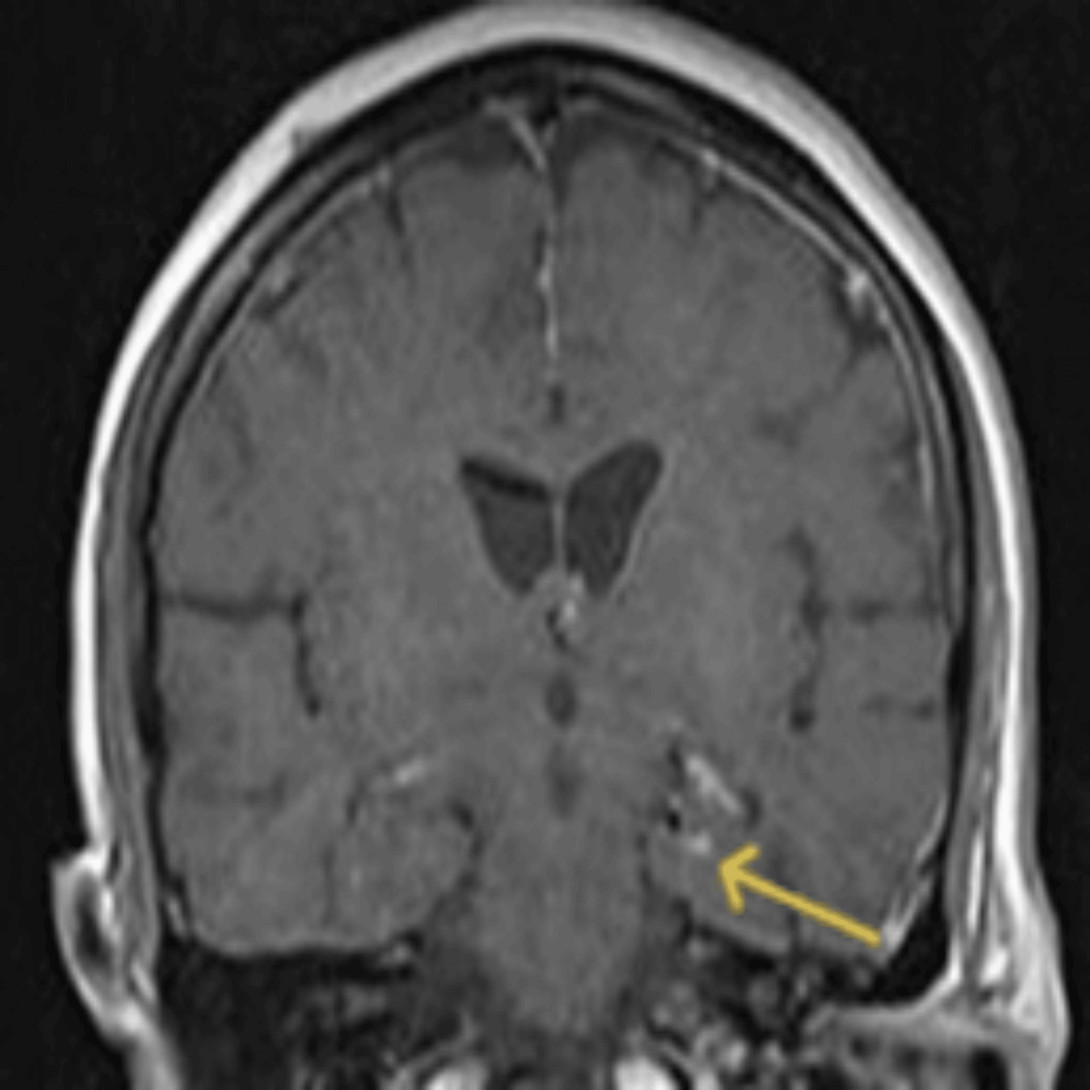
Coronal gadolinium-enhanced T1-weighted MRI The arrow reveals a spot-like enhanced lesion within the left temporal lobe area on the edge of the body of the left hippocampus of the brain.

Upon admission, both pre- and post-gadolinium brain and cervical spine magnetic resonance imaging (MRI) were performed using a Siemens Avanto 1.5 Tesla MRI scanner. The initial cervical MRI scan demonstrated thickening and enhancement of the entire left pharyngeal wall, extending from the level of the nasopharynx to the hypopharynx (cricoid cartilage), resulting in narrowing of the airway lumen. Moreover, enhancement was observed in the ipsilateral parapharyngeal space and submandibular region, affecting the submandibular gland (Figure 1) [6,7]. Brain MRI showed a high signal intensity area in the body of the left hippocampus on T2-weighted fluid-attenuated inversion recovery (FLAIR) imaging, along with a spot-like enhanced lesion within the aforementioned pathological area (Figures 2 and 3) [8,9]. These findings are indicative of herpetic encephalitis [4,8,10,11].

**Figure 4:**
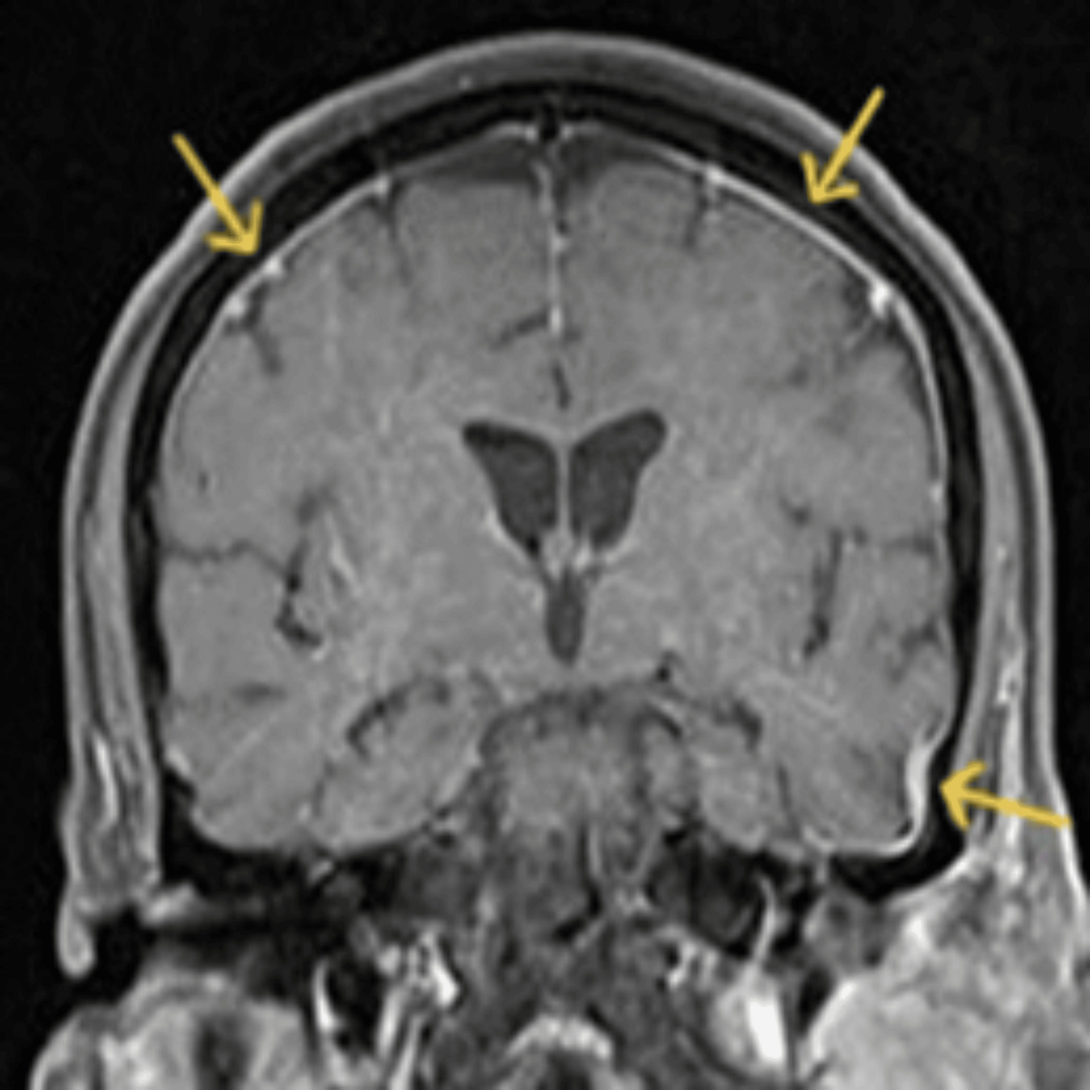
Coronal gadolinium-enhanced T1-weighted MRI Arrows depict the meningeal enhancement.

**Figure 5:**
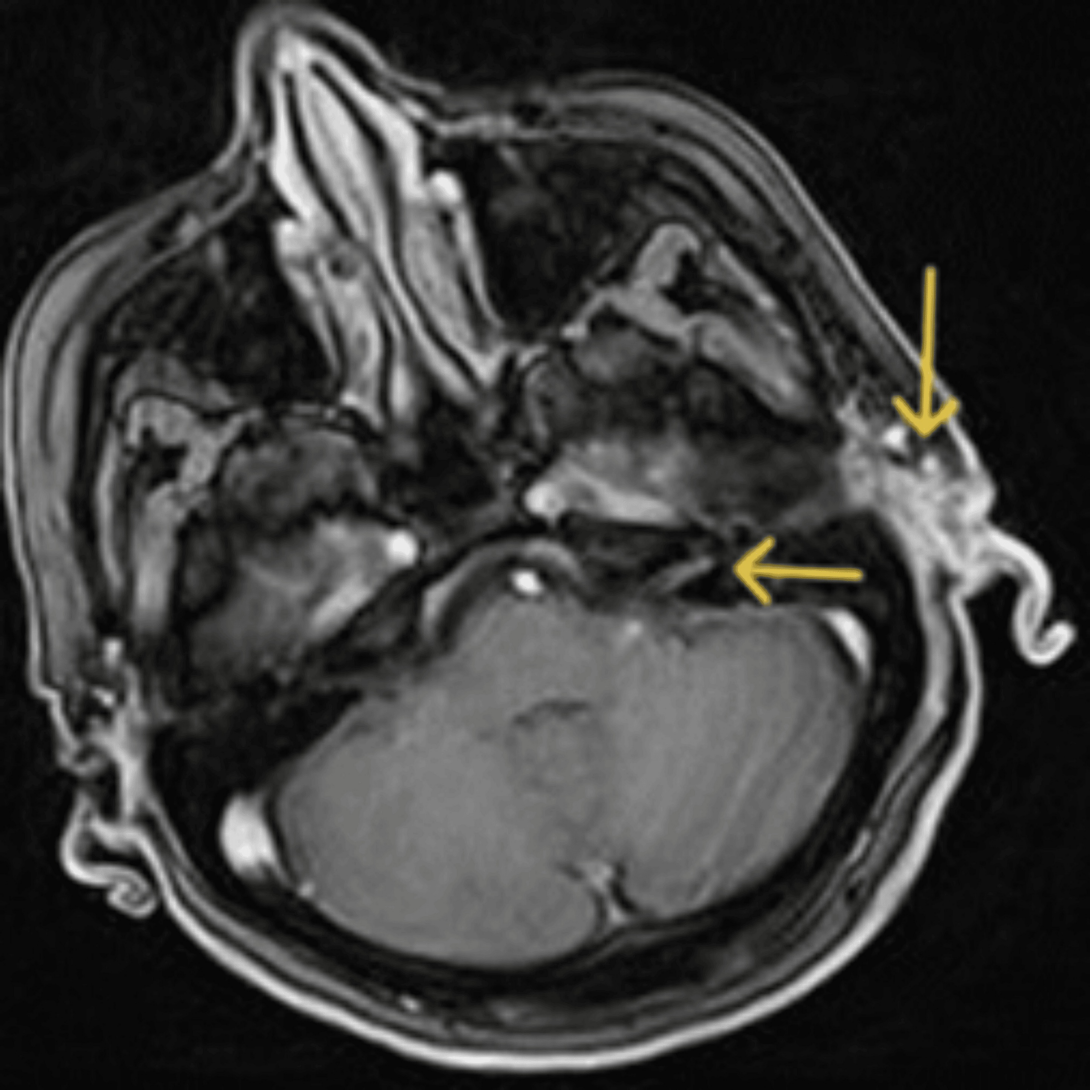
Axial gadolinium-enhanced T1-weighted MRI at the level of internal auditory canals Arrows indicate contrast enhancement within the left internal auditory canal and ipsilateral VII^th^ and VIII^th^ cranial nerves.

Post-gadolinium imaging demonstrated enhancement of the dura matter at the temporal lobe, indicative of meningitis (Figure 4) [8,12-14]. Contrast-enhanced T1-weighted MRI depicted enhancement of cranial nerves VII, VIII, IX, XII (Figure 5), consistent with the involvement of these nerves in varicella-zoster virus (VZV) infection [6,7,15]. Additional findings included fluid collections of approximately 5 mm in the retropharyngeal space up to the C7 level and the presence of left jugular lymph nodes up to 1 cm in the short axis [7].

Treatment and follow-up

After the initial laboratory tests and the first brain MRI scan, we initiated treatment with prednisolone (125 mg per day) and ocular protection to prevent ocular keratitis. After the established diagnosis of VZV and RHS, intravenous acyclovir (500 mg thrice daily) was administered for 32 days, with continuous renal function monitoring due to the drug's nephrotoxicity. Due to the patient's clinical deterioration, the findings from flexible nasendoscopy (white mucosa), the fever, and the increased respiratory secretions, antibiotics were administered, such as piperacillin/tazobactam (4 gr/0.25 gr three times daily) and ciprofloxacin (500 mg twice daily), while treatment was also supplemented with an antifungal agent (fluconazole). Prednisolone doses were gradually tapered and discontinued after 20 days. Respiratory function improved with budesonide, ipratropium, salbutamol, and bilevel positive airway pressure (BiPAP) breathing device. After two months of hospitalization, the patient's clinical presentation improved, although facial paralysis and dysphagia persisted. The patient was eventually discharged with a permanent tracheostomy, a nasogastric tube and entered a physical rehabilitation center. Six months later, she was discharged from the rehabilitation center, having regained swallowing function, restored mobility, and the ability to walk independently.

## Discussion

VZV is a neurotropic DNA virus that causes chickenpox during initial infection, then lies dormant in ganglion and may later reactivate to cause shingles. This virus can have a broad spectrum of different effects on the nervous system, affecting cranial nerves and spreading to the brainstem as well [8,9]. Immunosuppression is the most common cause of VZV reactivation [2,16]. Ramsay Hunt syndrome, or herpes zoster oticus, results from VZV infection of the geniculate ganglion - the sensory ganglion of the facial nerve (VII) [6]. RHS typically presents with painful vesicular eruptions in the external auditory canal and ipsilateral facial nerve palsy, which may occur before or after the onset of ear pain and vesicles [6].

In rare cases, RHS may manifest without the typical vesicular rash known as zoster sine herpete [5]. Vestibular symptoms, such as vertigo, tinnitus, and imbalance, dominate when the cochleovestibular nerve (VIII) is affected, often overshadowing cochlear symptoms like sensorineural hearing loss [3,17]. The cranial nerves most frequently involved are VII, VIII, IX, V, and X, while the involvement of cranial nerves I, II, III, IV, XI, and XII is less common [3,5,6]. Although life-threatening complications, such as multicranial polyneuropathy, meningitis, and encephalitis, are rare, they do occur and necessitate early diagnosis and prompt treatment with corticosteroids and antiviral therapy [4,5,18]. VZV DNA detection via PCR confirms the diagnosis of herpes zoster, though the clinical presentation combined with brain MRI findings often raises suspicion of RHS even before PCR confirmation [6,8,14,15,19]. Although MRI is not able to definitively diagnose herpes zoster, it remains a valuable diagnostic tool, particularly when complications from varicella zoster virus are suspected.

Our case is notable due to the unusual clinical manifestations; this is only the fourth reported case of RHS involving seven cranial nerves. Moreover, the patient exhibited lower cranial nerve palsy (IX, X, XI, XII), a rare manifestation, and is one of only five reported cases of hypoglossal nerve (XII) impairment during RHS [7,11,12]. Finally, this case is a rare case of RHS with such extensive cranial nerve involvement along with meningoencephalitis. Encephalitis itself is an unusual complication, occurring in only 20-28 cases per one million VZV infections [10]. Such cases highlight that varicella-zoster meningoencephalitis is an understated cause of central nervous system infections, often complicating RHS [10]. In our clinical examination, we meticulously avoided over-diagnosing glossopharyngeal, vagus, accessory, and hypoglossal impairment, with positive findings limited to instances of clear paresis or absent reflexes.

Brain MRI was highly important for our differential diagnosis as it revealed pathological signs in four out of seven affected cranial nerves. Enhancement of cranial nerves VII and VIII suggested herpes virus infection, as these are commonly affected in VZV reactivation [6,15,16]. Although identifying other cranial nerves in MRI is challenging due to their complex course and small size, in our case, cranial nerves IX and XII were observed. The larger size, specific brainstem origins, and foramina traversed by cranial nerves III, V, VII, VIII, IX, and XII make them more readily recognizable in MRI [7,20]. Thinner MRI sections facilitate nerve identification. In particular, neuropathy in cranial nerves VII and VIII may appear as nerve enhancement or thickening at the internal acoustic meatus, given their shared course, while cranial nerve VII may also show changes at the geniculate ganglion (Table 1) [15,20].

**Table 1:** The manifestations and MRI findings of each cranial nerve impairment Sources: [6-8,11,15]

Involved cranial nerves (CN)	Manifestations	MRI findings	
Trigeminal nerve (V)	tooth ache and reflex along the course of the trigeminal nerve, pain at the mandible		enhancement or thickening along the course of the nerve, especially in the region where the nerve exits the pons
Facial nerve (VII)	drop of lower lip angle, inability to close the eye, drop of the eyelid, loss of forehead wrinkles, loss of nasolabial fold		enhancement or thickening particularly at the geniculate ganglion or along its course through the temporal bone
Vestibulocochlear nerve (VIII)	imbalance, sensorineural hearing loss, tinnitus		enhancement or thickening at the internal acoustic meatus
Glossopharyngeal nerve (IX)	throat discomfort, dysphagia - inability to swallow, absent gag reflex		enhancement or thickening of the nerve or adjacent structures in the posterior cranial fossa
Vagus nerve (X)	semi-larynx paralysis with hoarseness, removal of the vomiting reflex		enhancement of thickening particularly in the posterior cranial fossa
Accessory nerve (XI)	weakness of the sternocleidomastoid and the trapezium muscle		changes in the nerve's course or branching within the neck
Hypoglossal nerve (XII)	tongue deviation to the normal side when removed from the mouth		enhancement or thickening

Ultimately, our patient developed meningoencephalitis, confirmed by MRI. Remarkable was the enhancement of the dura matter at the level of the temporal lobe, indicating meningitis. Additionally, the presence of spot-like enhancement around the left lateral ventricle's anterior horn on T2-weighted and FLAIR sequences, extending to the adjacent hippocampus, aligns with the hallmark of herpetovirus infections [4,8,10].

In conclusion, the combination of clinical manifestations with brain MRI features can strongly suggest RHS, even before PCR confirmation of VZV infection. In patients presenting with facial nerve palsy and painful eruptions in the ipsilateral ear, with or without vestibular or auditory symptoms, a brain MRI should be promptly considered. It is crucial to understand that MRI serves as the foundation for the diagnosis, even before PCR takes place, allowing for prompt treatment with acyclovir and prednisolone to prevent life-threatening complications [14]. 

## Conclusions

This case highlights the importance of clinical symptoms and brain MRI findings in the early diagnosis of VZV infection. A high index of suspicion for VZV infection is imperative, as the delayed diagnosis can cost valuable and vital time from immediate treatment and may subsequently lead to life-threatening complications. The role of MRI in the diagnosis of RHS associated with cranial polyneuropathy, meningitis, and/or encephalitis is highlighted. We recommend MRI in cases of facial nerve palsy and/or signs of vestibulocochlear nerve involvement, particularly when vesicular eruptions are present, as the detection of nerve enhancement raises suspicion of VZV infection; in such scenarios, combining MRI findings with clinical manifestations can expedite diagnosis, enabling early antiherpetic therapy and comprehensive management, which are critical for improving outcomes in these complex cases.
